# Phylogenetic relations and mitogenome‐wide similarity metrics reveal monophyly of *Penaeus* sensu lato

**DOI:** 10.1002/ece3.7148

**Published:** 2021-02-04

**Authors:** Vinaya Kumar Katneni, Mudagandur S. Shekhar, Ashok Kumar Jangam, Balasubramanian C. Paran, Ashok Selvaraj, Karthic Krishnan, Nimisha Kaikkolante, Sudheesh K. Prabhudas, Gopikrishna Gopalapillai, Vijayan K. Koyadan

**Affiliations:** ^1^ Nutrition Genetics and Biotechnology Division ICAR‐Central Institute of Brackishwater Aquaculture Chennai India; ^2^ Crustacean Culture Division ICAR‐Central Institute of Brackishwater Aquaculture Chennai India; ^3^ ICAR‐Central Institute of Brackishwater Aquaculture Chennai India

**Keywords:** average Aminoacid Identity, bayes tree, maximum Likelihood tree, penaeid phylogeny, penaeid taxonomy, penaeus sensu lato

## Abstract

Splitting of the genus *Penaeus* sensu lato into six new genera based on morphological features alone has been controversial in penaeid shrimp taxonomy. Several studies focused on building phylogenetic relations among the genera of *Penaeus* sensu lato. However, they lack in utilizing full mitochondrial DNA genome of shrimp representing all the six controversial genera. For the first time, the present study targeted the testing of all the six genera of *Penaeus* sensu lato for phylogenetic relations utilizing complete mitochondrial genome sequence. In addition, the study reports for the first time about the complete mitochondrial DNA genome sequence of *Fenneropenaeus indicus*, an important candidate species in aquaculture and fisheries, and utilized it for phylogenomics. The maximum likelihood and Bayesian approaches were deployed to generate and comprehend the phylogenetic relationship among the shrimp in the suborder, Dendrobranchiata. The phylogenetic relations established with limited taxon sampling considered in the study pointed to the monophyly of *Penaeus* sensu lato and suggested collapsing of the new genera to a single genus. Further, trends in mitogenome‐wide estimates of average amino acid identity in the order Decapoda and the genus *Penaeus* sensu lato supported restoration of the old genus, *Penaeus*, rather promoting the creation of new genera.

## Graphical abstract

The maximum likelihood and Bayesian phylogenetic inferences based on complete mitochondrial DNA genome sequences supported monophyly of the genus, *Penaeus* sensu lato. The mitogenome‐based similarity metrics estimated for between‐genera within a family and also for between‐species within a genus in the order Decapoda corroborated phylogenetic inferences drawn for *Penaeus* sensu lato.

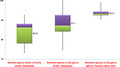

## 1 INTRODUCTION

Farming of penaeid shrimp has been crucial in many tropical developing countries by providing export revenue and rural livelihood support (Bush et al., 2010). In the past few decades, taxonomic nomenclature of the genus *Penaeus* sensu lato (*Penaeus* s.l.) underwent major revisions. Burkenroad (1934) divided 21 clearly characterized penaeid shrimp reported at that time into two subgroups, Division 1 and 2 based on few morphological traits. The Division 1 includes 12 shrimps exhibiting diagnostic characteristics such as adrostral carinae not extending to near the posterior margin of carapace, lacking postocular crest, rostrum bearing more than one ventral tooth, laterally unarmed telson, and ischium of the first pair of chelate legs armed with a spine whereas Division 2 includes nine species which are characterized by adrostral carinae extending almost to the posterior margin of carapace and the presence of postocular crest. He has also suggested that characters such as open thelycum, short adrostral carina, and ischial spine on the first cheliped are probably primitive characters for the genus *Penaeus*. A notable observation from his publication is that the word “subgenus” was also used in place of “Division” at some places. Subsequently, Kubo (1949) modified the classification of Burkenroad (1934) based on the presence or absence of hepatic carina and bifurcated Division 1 into two sub divisions. Later, Perez‐Farfante (1969) proposed four subgenera and added another character: thelycum type, in addition to those used by earlier workers to distinguish subgenera. The four subgenera are *Melicertus* [adrostral carina long & gastro‐frontal carina present], *Fenneropenaeus* [adrostral carina short, gastro‐frontal carina absent & hepatic carina ill‐defined/absent and closed thelycum], *Litopenaeus* [adrostral carina short, gastro‐frontal carina absent, hepatic carina prominent, petasma with short ventral costa & open thelycum], and *Penaeus* [adrostral carina short, gastro‐frontal carina absent, hepatic carina prominent, petasma with long ventral costa and closed thelycum]. In due course, two more subgenera were proposed which are *Marsupenaeus*, for shrimp with tube‐like thelycum (Tirmizi, 1971) and *Farfantepenaeus*, for American shrimp (Burukovsky, 1972).

There have been some debates regarding the creation of subgenera. Dall et al., (1990) showed concern in creating six subgenera in a genus containing only 29 species. To a large extent, the creation of subgenera had little effect on practitioners of shrimp farming or researchers as subgenera names are rarely used in scientific literature or in commerce (Flegel, 2007). However, in a relatively recent monograph, Pérez‐Farfante and Kensley (1997) elevated these subgenera into generic status, and this unilateral elevation has been controversial for the last two decades. Several publications (Baldwin et al., 1998; Dall, 2007; Flegel, 2008; Hurzaid et al., 2020; Lavery et al., 2004; Ma et al., 2009, 2011; Maggioni et al., 2001; McLaughlin et al., 2008) came out either in support or against the proposal of Pérez‐Farfante and Kensley (1997).

Looking into the events, it is evident that the terminology of “Division” which was started for the sake of convenience has become a taxonomic nomenclature (initially subgenus and then genus) which has been debated for the last two decades. The authors who have proposed a subgenus or genus have not considered phylogenetic evidence. Till date, the “Species fact sheets” published by Food and Agriculture Organization (FAO), Rome, uses the old genus nomenclature “*Penaeus*.” The new genera names have not been used in FAO webpages and fact sheets. This clearly indicates that the global research community is divided in adopting one genus versus six genera nomenclature. At this juncture, it has become very important to build true phylogenetic relations among the penaeid shrimp based on proper datasets and robust analytical methods.

Selecting morphological traits alone in taxonomic delineation has been extremely difficult or often misleading as symmetrical and simple diagnostic features are harder to achieve (Burkenroad, 1983). Genetic differences were observed between morphologically and ecologically similar species (Palumbi & Benzie, 1991). As morphological characters are susceptible to convergent evolution that would obscure the phylogenetic inferences (Hedges & Maxson, 1996), molecular approaches are widely used to resolve the taxonomic disagreement (Ma et al., 2011). In order to resolve the taxonomic confusion, several authors have reconstructed the phylogeny for species in the genus *Penaeus* s.l. using mitochondrial and nuclear DNA markers (Baldwin et al., 1998; Chan et al., 2008; Gusmão et al., 2000; Lavery et al., 2004; Ma et al., 2009, 2011; Quan et al., 2004; Voloch et al., 2005; Wang et al., 2004). Although the science of taxonomy was revolutionized by the DNA barcoding system, it has been argued that the sequence may be, at least in some cases, uninformative if only one portion of genome is used (Galtier et al., 2009). In traditional mitochondrial taxonomy, only partial sequence of one gene has been used, and it is often insufficient to resolve the relationship when radiation is very rapid (Morin et al., 2010). The resolution of phylogeny could be increased and refined by increasing the amount of sequence data (DeFilippis & Moore, 2000; Rokas & Carroll, 2005). Therefore, the complete mitochondrial genome is found to be an ideal marker for phylogeny, population genetic diversity, and maternal inheritance (Ma et al., 2013, 2015). Even with complete mitogenome sequences, insufficient taxon sampling could hinder resolution of certain interrelationships as shown with infraorders in the order, Decapoda (Tan et al., 2019). However, in all the previous analyses conducted utilizing complete mitogenomes of *Penaeus* s.l., shrimp in the subgenus *Melicertus* were missing as sequence data are not available. Our study included the representative shrimp from the subgenus *Melicertus* (Zhong et al., 2018) and also the accession of *Fenneropenaeus indicus* which is reported in this study.

Setting aside the controversies of one genus versus six genera taxonomic nomenclature for penaeid shrimp, the phylogenetic relations among them based on complete mitochondrial genomes are of paramount interest to researchers in the field. The main objective of this paper is to determine whether the controversial assignment of genera in *Penaeus* s.l. based on the morphology alone (Pérez‐Farfante & Kensley, 1997) is consistent with the phylogenetic affiliation based on complete mitochondrial genome. In addition, mitogenome‐based similarity metrics have been explored for the first time as additional metrics to comment on the relevance of six genera in *Penaeus* sensu lato.

## 2 MATERIALS AND METHODS

### 2.1 Sequence datasets

The complete mitochondrial DNA genomes in suborder Dendrobranchiata including ten of the shrimp in *Penaeus* sensu lato were used for phylogenetic analysis (Table 1). The suborder Dendrobranchiata has two superfamilies, Penaeoidea and Sergestoidea. The Penaeid shrimp, whose taxonomy is being addressed in this paper, belongs to the superfamily, Penaeoidea. Therefore, the sequence of *Sergia lucens* from the superfamily, Sergestoidea was kept as outgroup for phylogenetic analysis in this study. The materials and methods used for assembling complete mitochondrial DNA of *F. indicus* have been given in Appendix A. Two datasets were used for phylogenetic reconstruction of *Penaeus* s.l. viz. combined sequences of all 13 protein‐coding genes and combined sequences of 2 rRNA genes. Initially, individual gene sequences were aligned in MAFFT v7.305b (Katoh & Standley, 2013) following L‐INS‐i strategy setting maximum iterations to 1,000. The individual gene alignments were examined using Guidance2 tool (Sela et al., 2015) to identify the positions in the alignment that have poor alignment confidence scores (<0.93). Those positions with poor confidence scores, present in less than 25% accessions and some additional positions to keep triplet codon structure in case of protein‐coding genes were removed. Then, individual gene alignments were concatenated to obtain final alignment for phylogenetic analyses. In‐depth details about preparation of sequence alignments have been mentioned in Appendix B.

**TABLE 1:** Complete mitochondrial DNA genomes of the Super family, Penaeoidea, used in this study

Sl. No.	Organism	Genbank_Id
1	*Aristeomorpha foliacea*	MG582604
2	*Aristeus virilis*	MG582605
3	*Benthonectes filipes*	MF379624
4	*Farfantepenaeus californiensis*	EU497054
5	*Fenneropenaeus chinensis*	DQ518969
6	*Fenneropenaeus indicus*	KX462904
7	*Fenneropenaeus merguiensis*	KP637168
8	*Fenneropenaeus penicillatus*	KP637169
9	*Gennadas parvus*	MF379623
10	*Gordonella aff. paravillosa ZS‐2018*	MF379625
11	*Hymenopenaeus neptunus*	MF379622
12	*Litopenaeus stylirostris*	EU517503
13	*Litopenaeus vannamei*	EF584003
14	*Marsupenaeus japonicus*	AP006346
15	*Metapenaeopsis barbata*	MG833230
16	*Metapenaeopsis dalei*	KU050082
17	*Metapenaeus affinis*	MG815825
18	*Metapenaeus ensis*	KP637170
19	*Parapenaeopsis hardwickii*	KU302814
20	*Parapenaeopsis hungerfordi*	MG873460
21	*Melicertus latisulcatus*	MG821353
22	*Penaeus monodon*	AF217843
23	*Pleoticus muelleri*	MH500232
24	*Sergia lucens*	LC368254
25	*Sicyonia lancifer*	MF379620
26	*Sicyonia parajaponica*	MF379619
27	*Solenocera crassicornis*	MF379621

In addition, two more datasets containing all protein‐coding and rRNA gene sequences of the 10 complete mitochondrial genomes of *Penaeus* s.l. were prepared and utilized to study phylogenetic relations. The *Metapenaeus ensis*, a species in the superfamily, Penaeidae does not belong to the *Penaeus* s.l., but has been used as outgroup for building these phylogenetic trees. Detailed alignment procedures were mentioned in Appendix B.

### 2.2 Maximum likelihood tree

The protein‐coding and rRNA genes datasets were used to build Maximum Likelihood (ML) tree in RAxML version 8.2.9 (Stamatakis, 2014) with GTRGAMMAI model (GTR substitution model with gamma distributed rates and a proportion of invariant sites). For protein‐coding genes, the best fit partitioning schemes obtained using PartitionFinder v2.1.1 (Guindon et al., 2010; Lanfear et al., 2017) were also defined for building ML tree. A hundred multiple searches were made to find the best starting tree with a random seed 12,345. Then, nodal support for the tree with best likelihood was obtained with 1,000 bootstrap replications.

### 2.3 Bayesian tree

The datasets were also subjected to Bayesian inference analysis in MrBayes 3.2.6 (Huelsenbeck & Ronquist, 2001). For protein‐coding genes, the best fit models and partitioning schemes are defined as per the results from PartitionFinder v2.1.1. For rRNA genes dataset, GTRGAMMAI model was employed to build Bayes tree considering each rRNA gene sequence as a partition. The underlying evolutionary process was assumed to be different for each gene and hence a separate set of parameters was estimated for each gene partition. Two simultaneous but completely independent runs that start from different random trees were executed with four chains for 10 million generations while sampling trees every 100 generations and calculating convergence statistics every 1,000 generations. First, 25% of samples were discarded and hence not included for calculating summary statistics. The maximum standard deviation of split frequencies and potential scale reduction factor, PSRF (Gelman & Rubin, 1992) were used to check the convergence of runs. The same was performed by also examining the trace files in Tracer v1.6 (Rambaut et al., 2014).

### 2.4 Genome‐based similarity metrics

A genome‐wide similarity metric, the Average Aminoacid Identity (AAI) was estimated among the species in each genus of the order “Decapoda” where complete mitochondrial genome is available for at least two species. The AAI values were estimated using the AAI calculator (Rodriguez‐R & Konstantinidis, 2016) maintained at Kostas lab.

## 3 RESULTS

### 3.1 Mitochondrial genome of *F. indicus*

**FIGURE 1:**
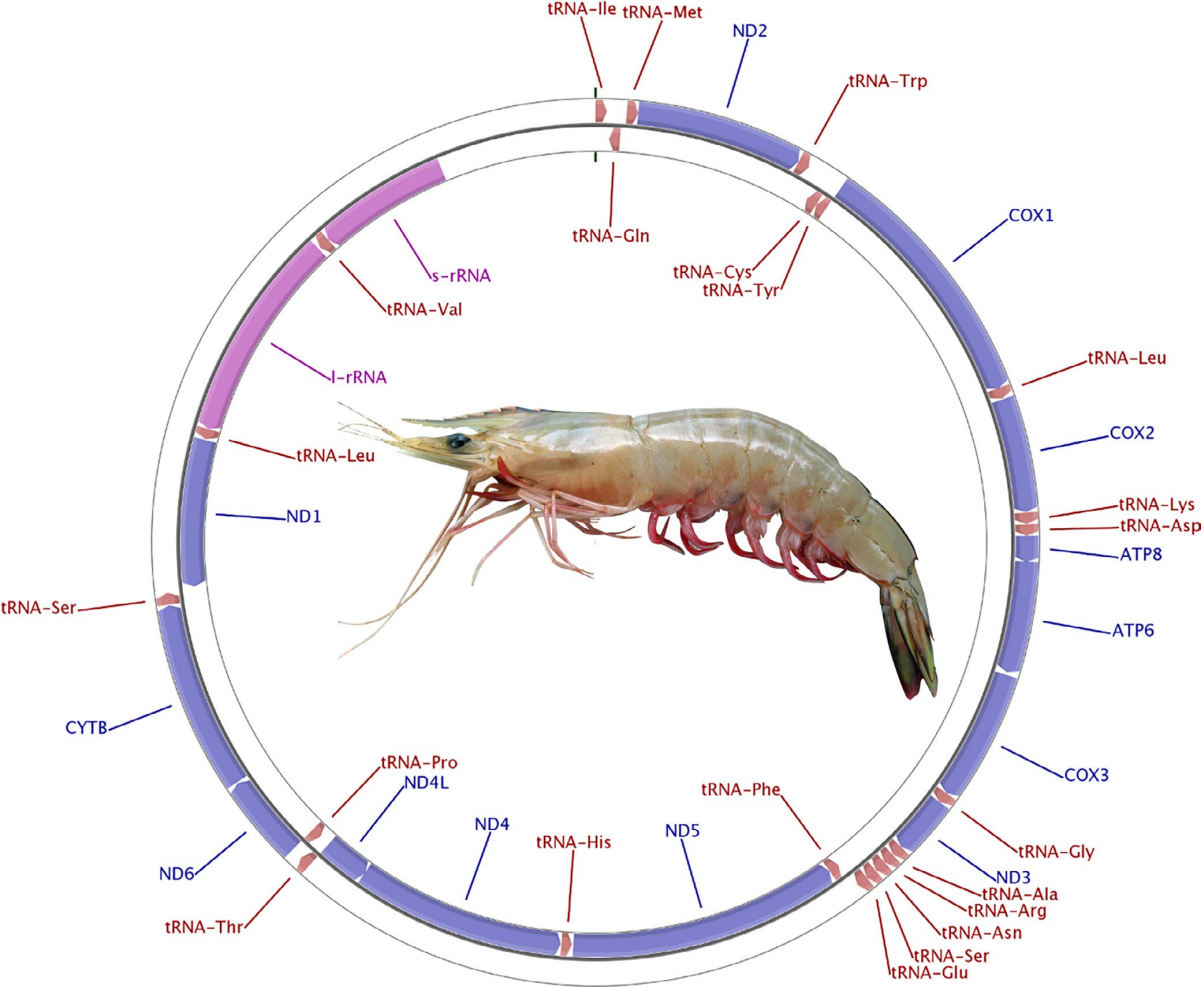
Mitochondrial DNA genome of *Fenneropenaeus indicus*. The circular plot depicts the predicted protein‐coding genes (blue), rRNA gens (pink) and tRNA genes (brown) in the genome. The predicted genes on plus and minus strand are shown in outer and inner rings, respectively. At the center of the circular plot is an image of the *F. indicus*

The specimen used for generating mitogenome sequence of *F. indicus* was confirmed for the species based on the partial sequence of the barcoding gene, Cytochrome c Oxidase I (Appendix A). The features of mitochondrial genome of *F. indicus* have been depicted in Figure 1 and presented in detail in Appendix A. The arrangement of genes in mitochondrial genome is similar in all species of *Penaeus* sensu lato. The predicted secondary structures of all tRNAs are depicted in Figure A2 (see Appendix A).

### 3.2 Maximum likelihood trees

**FIGURE 2:**
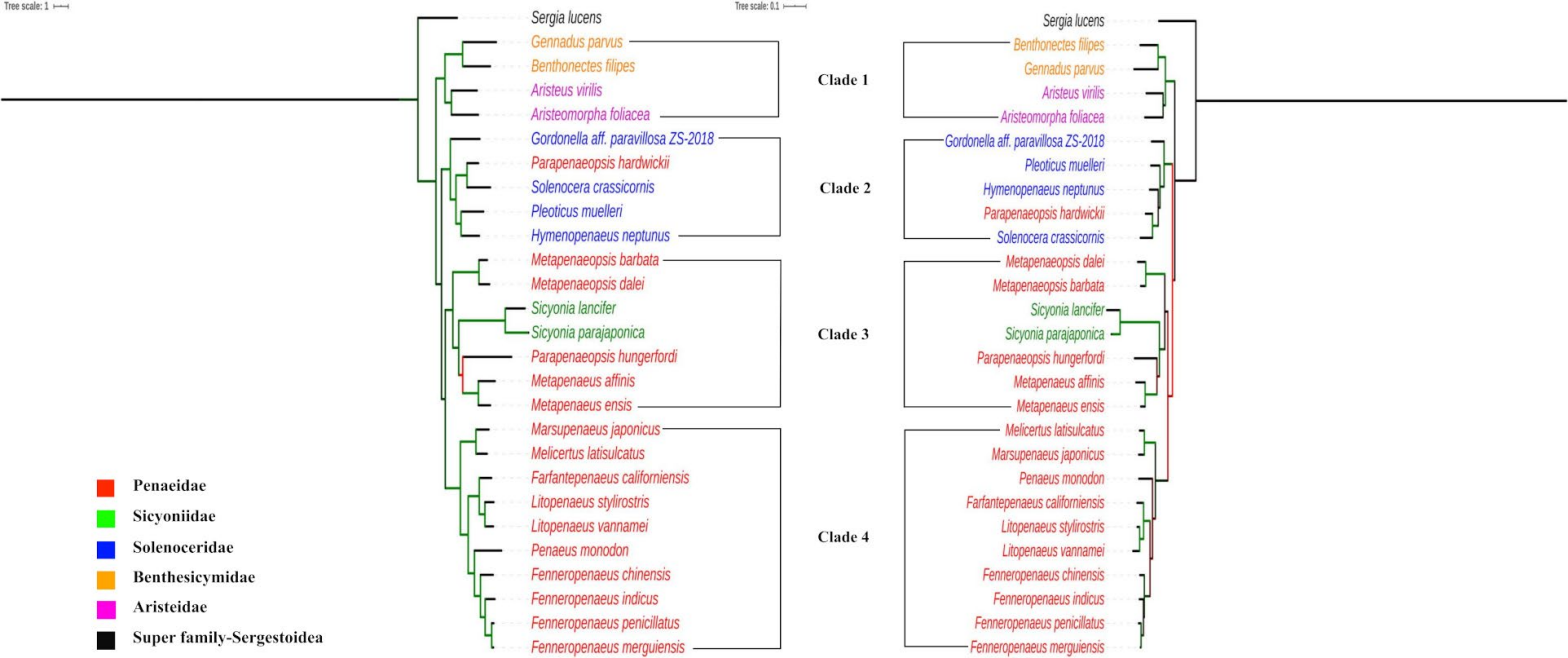
Phylogenetic trees for protein‐coding genes (left) and rRNA genes (right) derived by maximum likelihood method. The branches are colored based on the bootstrap values (≥90 = green; ≥70 and <90 = black; and <70 = red)

The detailed results of analyses indicating the best partitioning schemes and the evolutionary models for protein‐coding sequence datasets have been given in Appendix B. The Maximum Likelihood (ML) trees built for shrimp in the superfamily, Penaeoidea with protein‐coding and rRNA genes datasets have been depicted in Figure 2. The random seed used for finding the best tree as well as for conducting bootstraps was 12,345. Both the protein‐coding and rRNA genes datasets are in perfect agreement on structure of phylogenetic tree. The shrimp of superfamily Penaeoidea are clustered into 4 distinct clades. The clade 1 has shrimp that belongs to family Benthesicymidae (benthesicymid shrimp) and family Aristeidae (gamba shrimp). The clade 2 consists majorly shrimp of family Solenoceridae (solenocerid shrimp) and one shrimp (*Parapenaeopsis hardwickii*) from family Penaeidae. The shrimp of family Sicyoniidae (rock shrimp) and Penaeidae (penaeid shrimp) are clustered in clade 3. All the shrimp clustered in clade 4 belong to family Penaeidae. Interestingly, all the penaeid shrimp in clade 4 belong to the genus, *Penaeus* sensu lato. All the four clades are supported with high (> 90%) bootstrap values.

The clade 4 shrimp could further be clearly demarcated to three subclades. One subclade has grooved shrimp (*Marsupenaeus* and *Melicertus*), one subclade has shrimp from American waters (*Litopenaeus* and *Farfantepenaeus*), and the other subclade has shrimp from Asian waters (*Penaeus* and *Fenneropenaeus*). Interestingly, all nodes in clade 4 of ML tree are completely supported in all bootstrap replicates. Of two shrimp in genus *Parapenaeopsis*, a penaeid shrimp, the *P. hardwickii* is clustered with solenocerid shrimp, and the *P. hungerfordi* is clustered with rock shrimp.

For *Penaeus* s.l., the ML tree established a close sister relation between *F. penicillatus*, and *F. merguiensis* for shrimp in Asian waters and between *L. vannamei* and *L. stylirostris* for shrimp in American waters. The species of these genera formed monophyletic groups which are correlated to geography.

### 3.3 Bayesian trees

**FIGURE 3:**
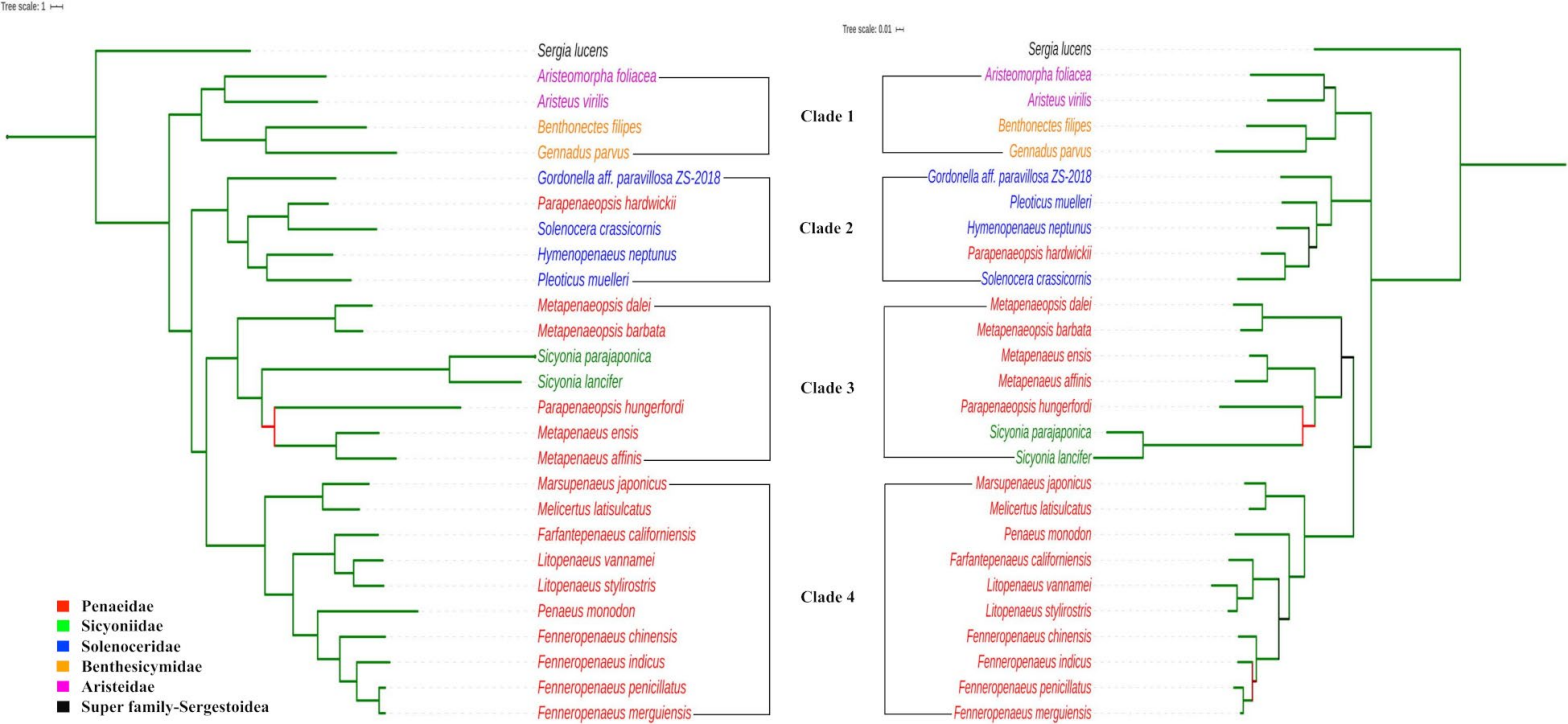
Phylogenetic trees built for protein‐coding genes (left) and rRNA genes (right) derived by Bayesian method. The branches are colored based on the posterior probability values (≥0.9 = green; ≥0.7 and <0.9 = black; and <0.7 = red)

The maximum standard deviation of split frequencies and potential scale reduction factor, PSRF (Gelman & Rubin, 1992) were used to check the convergence of Bayesian runs. The maximum standard deviation of split frequencies was less than 0.01 for all the datasets indicating convergence of two runs. Detailed results of the Bayesian analysis have been given in Appendix B. The Bayesian trees built with protein‐coding and rRNA genes datasets for Penaeoid shrimp have been depicted in Figure 3. The Bayes trees are corroborating the results obtained in ML trees and did not contradict any inference made on ML trees. Like ML tree, Penaeoidean shrimp are clustered into four similar clades. Most of the nodes in both the Bayes trees have 100% bootstrap support values.

### 3.4 Genome‐wide similarity metrics

Wherever mitochondrial genome data were available for a minimum of two species under a genus, the average amino acid identity (AAI) was estimated between all the species in each genus of order, Decapoda (Appendix C and D). For each genus, the minimum and maximum AAI estimate obtained are given as ranges in Appendix D. Wherever complete mitochondrial DNA genome is available for only two species in a genus, the single estimate of AAI between them is given. For 45 different genera, the range of genome‐wide estimates obtained would establish the general trend of estimates for order Decapoda. The aim was to compare the within‐genus trend in the order Decapoda with the estimates in the genus, *Penaeus*. As suggested by a reviewer, we have also estimated the genome‐wide similarity metrics for between‐genera (within each family) in the order, Decapoda. Here, for each genus, one representative species was randomly used to get between‐genus similarity metrics in each family of Penaeioidea. The objective is to examine the general trend of similarity metrics at between‐genus level in relation to between‐species level.

Among species in genus *Penaeus* s.l., the highest AAI was estimated between *F. penicillatus* and *F. merguiensis* (98.95) followed by that between *L. vannamei* and *L. stylirostris* (98.09). The lowest AAI estimate was observed between *F. californiensis* and *P. monodon* (90.57). The highest AAI in order Decapoda was estimated (99.87) in genus *Helice* between *H. latimera* and *H. tientsinensis*. The lowest AAI of 77.95 in order Decapoda was estimated in genus *Engaeus* between *E. lengana* and *E. lyelli*. Except for few estimates in the genera, *Engaeus*, *Palaemon, Panulirus,* and *Typhlatya*, all others are greater than 80.0 (see Appendix C and D).

## 4 DISCUSSION

The gene order in *F. indicus* mitochondrial genome is similar to other shrimp genomes considered in our study. The length‐varying part of genome viz. the control region, is 1,001 bp long in Indian white shrimp and varies from 989 to 1,001 bp in *Penaeus* sensu lato. The positioning of control region between *srRNA* and *tRNA^Ile^* is a typical feature of Arthropods (Miller & Austin, 2006). The incomplete stop codons observed for six protein‐coding genes in *F. indicus* mitogenome is a common feature in animal mitochondrial genes where the stop codons are created by posttranscriptional polyadenylation (Ojala et al., 1981). The overlap observed between reading frames of ATP8 and ATP6 genes and that of ND4 and ND4L genes is a common phenomenon in crustacean species.

All previous attempts to construct phylogenetic relations among species of genus *Penaeus* s.l. considered only one/few of the mitochondrial or nuclear DNA genes (Lavery et al., 2004; Ma et al., 2011; Maggioni et al., 2001). For the first time, complete mitochondrial genomes of all the subgenera of genus *Penaeus* s.l. have been used in this study. Two tree building methods (Maximum Likelihood and Bayesian) involving all protein‐coding and rRNA genes improved the credibility and robustness of phylogeny. The present study provides the first phylogenomic evidence to support the monophyletic origin of genus *Penaeus* sensu lato. The most recent study on the phylogeny of *Penaeus* s.l (Ma et al., 2011) used three nuclear and two mitochondrial genes and concluded that this genus is monophyletic. The present study confirms and extends further to these findings.

Both the tree building methods on either of protein‐coding and rRNA genes datasets generated trees where shrimp of superfamily Penaeoidea are clustered into 4 distinct clades. The gamba shrimp and benthesicymid shrimp formed a single clade, thus supporting the previous works (Ma et al., 2009; Robalino et al., 2016; Wolfe et al., 2019) on monophyletic status for family Aristeidae and Benthesicymidae. Of four clades, only the clade 4 has species representing single family whereas all other clades have species from more than one family. Interestingly, all the shrimp in clade 4 belong to a single genus, *Penaeus* sensu lato. The subclades observed in clade 4 holding shrimp from American waters and Asian waters were also reported in other phylogenetic trees (Cheng et al., 2018; Lavery et al., 2004; Zhong et al., 2019). Each of the three subclades represents shrimp of two different genera, *Penaeus* and *Fenneropenaeus*; *Litopenaeus* and *Farfantepenaeus*; and *Marsupenaeus* and *Melicertus*. The protein‐coding and rRNA genes indicated a clear trend in phylogenetic relations in these subclades. The same three subclades were also constructed for penaeid shrimp in phylogenetic trees derived from combined sequence datasets consisting of two mitochondrial (COI, 16S rRNA) and two nuclear (sodium potassium ATPase alpha subunit, phosphoenolpyruvate carboxykinase) genes (Hurzaid et al., 2020). These subclades were also clearly constructed when phylogenetic relations are drawn with accessions of *Penaeus* sensu lato alone (Appendix E). Starting with closely related sister taxa, the relations in Asiatic shrimp from closest to farthest could be summarized as [((((*F. penicillatus* and *F. merguienesis*), *F. indicus*), *F. chinensis*), and *P. monodon*)]. Similarly, the relations among American shrimp could be established as [(*L. vannamei* and *L. stylirostris*), *F. californiensis*]. A close sister relation was reconstructed between *L. vannamei* and *L. stylirostris* in ML and Bayes trees which got almost 100% support in bootstrap replications and posterior probability values, respectively. The same nodes were recovered during tree reconstruction with random datasets.

For *Penaeus* s.l., the most closely related species are *F. penicillatus* and *F. merguienesis,* and perhaps these Asian shrimps are the most recently diverged species. Even the AAI estimates are higher between these species. The *M. japonicus* and *M. latisulcatus* formed a separate subclade and might be the early diverged shrimp in *Penaeus* sensu lato. Both these shrimp have consistently lower similarity indices with other shrimp.

**FIGURE 4:**
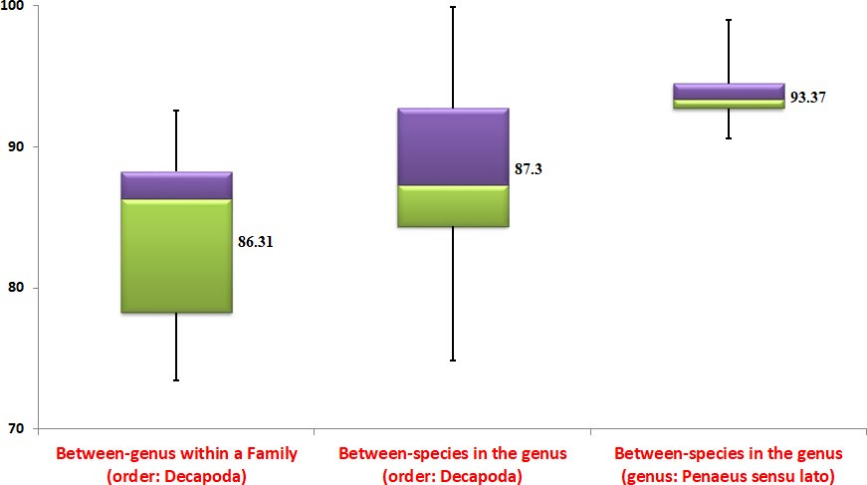
The box and whisker plot of AAI estimates obtained for between‐genera within a family; between‐species within a genus and between‐species in the genus *Penaeus* sensu lato in the order Decapoda. The values plotted are minimum, 25th percentile, median, 75th percentile, and maximum. The numerical value at each box is the median AAI estimate

The ambiguity about the monophyletic status for *Penaeus* s.l. still persists as various phylogenetic studies conducted in the past gave mixed inferences. The current study clearly indicated monophyletic status for *Penaeus* s.l. based on trees constructed on maximum likelihood and Bayes principles using all protein‐coding and rRNA genes in the mitochondrial DNA genome. In this scenario, further statistics testing the relationships among penaeid shrimp would add value to the inferred phylogenetic relations. In that line, we have used AAI estimates among mitochondrial DNA genomes to comment further on phylogenetic relations. Initially, we have established the trends in AAI estimates for between‐genus and between‐species comparisons in the order, Decapoda. Then, the AAI estimates among the species in *Penaeus* s.l. were compared to this established trend. A general trend that could be deduced from box plots of AAI estimates presented in Figure 4 is that the between‐species estimates tend to be higher than the between‐genus estimates. The between‐species AAI estimates for 45 genera in the order Decapoda have a median of 88.57. The between‐genera AAI estimates for 11 families in the order Decapoda have a median of 86.32. In *Penaeus* sensu lato, 45 AAI estimates were obtained among 10 species. The median of these 45 estimates is 93.37 which is higher than the median in the order, Decapoda. All the AAI estimates obtained among species of *Penaeus* s.l. are higher than the median estimate obtained for between‐species of all genera in the order, Decapoda. Though there are no set standard values for AAI estimates to fix different species in a genus, it is expected that the species of same genus have high AAI estimates. The between‐species AAI estimates across all the genera in the order, Decapoda indicated that the estimate tends to be higher (>80) in most of the cases. Only 18 out of 405 estimates were less than 80. Therefore, the general trend establishes higher AAI estimates between species of the same genus. When it comes to shrimps of *Penaeus* s.l., the AAI estimates are all above 90 (Figure 4 and Appendix C). The high estimates of AAI within *Penaeus* s.l. are consistent with comparisons of congeneric species in other genera of the order, Decapoda.

While splitting the genus *Penaeus* s.l. into subgenera and later into genera (Perez‐Farfante, 1969; Pérez‐Farfante & Kensley, 1997) the key biological evidence considered had been the shape of external genitalia of females, the thelycum (open vs. closed), a structure which is used to store sperm prior to spawning (Bauer, 1998). It was argued (McLaughlin et al., 2008) that these differences provide ample evidence to separate *Penaeus* s.l. into genera in the proposed taxonomy of Pérez‐Farfante and Kensley (1997). Further, the open thelycum was considered to be an ancient character, and it allowed them to suggest that the genus *Penaeus* s.l. originated first in the western hemisphere (Burkenroad, 1934). However, other researchers (Ma et al., 2011) opined that this hypothesis does not reflect the true evolutionary trend. The divergence in reproductive structure and behavior may cause strong selection pressure as difference in the reproduction related structure can facilitate prezygotic isolation between species, and therefore, it may evolve faster than postzygotic isolation (Mendelson, 2003). According to the molecular data, sister clade of *Farfantepenaeus* and *Litopenaeus* that inhabit the American waters may have evolved sympatrically owing to the strong sexual selection that drives rapid development of prezygotic isolation (Ma et al., 2011). They further concluded that external morphology of reproductive organ is a derived trait and that does not reflect true phylogenetic relationship. Our molecular phylogenetic data reveal that *L. vannamei, L. stylirostris*, and *F. californiensis* have been monophyletic, and this observation is in agreement with other findings (Lavery et al., 2004; Ma et al., 2011; Maggioni et al., 2001).

The second important morphological trait used for phylogeny and classification of genus *Penaeus* s.l. has been the presence or absence of the adrostral groove. The grooved shrimp have a long and distinct adrostral groove almost along the entire dorsal carapace (Burkenroad, 1934; Burukovsky, 1972; Kubo, 1949; Perez‐Farfante, 1969; Tirmizi, 1971) and this group includes the genera: *Marsupenaeus*, *Melicertus,* and *Farfantepenaeus*. The nongrooved shrimps are again subdivided into taxa with hepatic ridge (*Litopenaeus* and *Penaeus* sensu stricto) and without hepatic ridge (*Fenneropenaeus*). Although Ma et al., (2011) have not explicitly stated the characters used for assigning the six genera, the overall similarities based on the morphological traits formed the basis of their classification. However, our study using mitogenome data does not reflect the evolutionary partitions within the genus *Penaeus* sensu lato. Although it is hard to deny the importance of morphological data in phylogeny reconstruction (Wiens, 2004), it is inherently problematic to use morphological data (Scotland et al., 2003). This is particularly true among the species of genus *Penaeus* s.l. as most of the diagnostic characters of the species are subtle and have less resolution (Flegel, 2007). As morphological diagnostic traits of this genus are unsupportive to provide the true evolutionary trend, it becomes imperative to use molecular data. Our phylogenomic analysis strongly supports the monophyly of *Penaeus* s.l. in accordance with previous studies that used mitochondrial as well as nuclear genes (Baldwin et al., 1998; Lavery et al., 2004; Ma et al., 2009, 2011). Further, our phylogenomic studies are in concordance with the biogeographic provinces defined for marine fauna (Baldwin et al., 1998). As expected from the pan tropical distribution of the genus *Penaeus* s.l., unambiguous biogeographical clades were observed. For example, clustering of the three American shrimps (*L. vannamei*, *L. stylirostris,* and *F. californiensis)* and the four Asiatic white shrimp (*F. merguiensis*, *F. penicillatus, F. indicus,* and *F. chinensis*).

The ultimate goal of taxonomic science is to have taxonomy where both morphological and molecular data are in agreement (Dall, 2007) and to develop a natural system that reflects the phylogenetic relationship (Schram & Ng, 2012). Morphological differences separating the subgenera/genera in the genus *Penaeus* s.l. have also been extremely minor (Dall, 2007), and most lay people would not be able to differentiate them even at species level (Flegel, 2008). Perez‐Farfante (1969) has also opined that the creation of subgenera based on morphological characters is arranged solely for convenience of their recognition and no phylogenetic inferences should be drawn from it. Later when Pérez‐Farfante and Kensley (1997) proposed the new binomial revision, they demonstrated the same morphological/anatomical differences used for the erection of subgenera (Flegel, 2007). Recently, Ma et al., (2011) opined that revision of genus proposed by Pérez‐Farfante and Kensley (1997) were not in a strict cladistics sense. Taxonomic revision based on a gene sequence analysis of a single gene also raises concern by previous workers (Baldwin et al., 1998; McLaughlin et al., 2008). Earlier, Peregrino‐Uriarte et al., (2009) established monophyly of genus *Penaeus* s.l. based on phylogenomic analysis using maximum parsimony and maximum likelihood methods on nucleotide and amino acid datasets. In the present study, we have used complete mitochondrial genome of all the representative subgenera/genera of *Penaeus* s.l. and confirm that *Penaeus* s.l. is monophyletic group. However, the study suffers from limited taxon sampling as mtDNA genome sequences are not available for all genera of the family, Penaeidae.

## 5 CONCLUSIONS

The main purpose of this study was to determine whether the controversial genera assignment in *Penaeus* s.l. is consistent with the phylogenetic affiliation based on complete mitochondrial genome and to utilize mitogenome‐based similarity metrics as additional statistics to comment on phylogenetic relations. For the first time, representative shrimp of all the six genera were tested for phylogenetic relations. The complete mitochondrial genome of a culture‐relevant shrimp species, *F. indicus*, is generated and utilized in the study. The phylogenetic analyses clearly demonstrated monophyletic status and aptness of single genus nomenclature for shrimp in *Penaeus* sensu lato despite minor morphological differences. The trend of genome‐wide similarity indices in decapod genera corroborated the findings from phylogenetic analyses. The results are in concordance with Ma et al., (2011) in dismissing six genera nomenclature and favors reinstating the old genus *Penaeus*. Therefore, we recommend that the six genera scheme proposed by Perez Farfante and Kensley can be overturned and single genus status for *Penaeus* sensu lato may be reinstated. The ultimate goal must be taxonomy where both morphological and molecular data are congruent.

## CONFLICT OF INTEREST

The authors declare that there is no conflict of interest.

## AUTHOR CONTRIBUTIONS

**Vinaya Kumar Katneni:** Conceptualization (equal); Data curation (equal); Formal analysis (equal); Software (equal); Writing‐original draft (equal). **Mudagandur S. Shekhar:** Conceptualization (equal); Funding acquisition (equal); Project administration (equal); Writing‐review & editing (equal). **Ashok Kumar Jangam:** Data curation (equal); Formal analysis (equal); Software (equal); Writing‐review & editing (equal). **Balasubramanian C. Paran:** Investigation (equal); Writing‐original draft (equal). **Ashok Selvaraj:** Data curation (equal); Formal analysis (equal); Software (equal). **Karthic Krishnan:** Formal analysis (equal); Visualization (equal). **Nimisha Kaikkolante:** Formal analysis (equal); Visualization (equal). **Sudheesh K. Prabhudas:** Formal analysis (equal); Visualization (equal). **Gopikrishna Gopalapillai:** Writing‐review & editing (equal). **Vijayan K. Koyadan:** Conceptualization (equal); Project administration (equal); Resources (equal); Writing‐review & editing (equal).

## Supporting information

Appendix S1

## Data Availability

The mitochondrial genome sequence of *F. indicus* generated in this study is deposited at Genbank with accession number, KX462904.
