# Supplementary material for: Phylogenetic relations and mitogenome‐wide similarity metrics reveal monophyly of Penaeus sensu lato
Source: Ecol Evol. 2021 Feb 4;11(5):2040–9. doi: 10.1002/ece3.7148 (PMC7920775; doi:10.1002/ece3.7148)
Supplement: Supplementary file 1 — Appendix S1 [file ECE3-11-2040-s001.docx]

**APPENDIX S1**

**APPENDIX A: The mitochondrial DNA genome of *F. indicus***

**Accession of Indian white shrimp**

A specimen of Indian white shrimp was collected from Chennai coast (N 13° 7' 30.4104", E 80° 17' 43.9512) of India. The identity of the specimen was confirmed in two stages, first at morphological level by examining the external body features specific to the species and secondly at molecular level based on the barcoding gene, Cytochrome c Oxidase I (COI). Taking cue from the quality control guidelines published for validation of new mitogenomes by Botero-Castro et al., [Botero-Castro et al., 2016], we constructed a phylogenetic tree based on the partial COI gene sequence of the specimen and of the other *Penaeus* species present in NCBI and the BOLD database (<http://www.boldsystems.org/> - taken accessions with Barcode Index Numbers) to confirm the morphological identification. In total, about 328 accessions from complete mitochondrial genome submissions at NCBI and BOLD database were used for tree building. Initially, all the sequences were aligned using MAFFT v7.305b [Katoh et al., 2013] following L-INS-i strategy which is iterative refinement method incorporating local pairwise alignment information. The maximum iterations option was set to 1000. The sequence alignment was also analysed using Guidance2 tool [Sela et al., 2015] to identify the positions in the alignment that have poor alignment confidence scores (< 0.93). The final consensus alignment was used to build Maximum Likelihood (ML) tree in RAxML version 8.2.9 [Stamatakis et al., 2014] with GTRGAMMA model, random seed value of 12345 keeping *Metapenaeus ensis* as outgroup. The confidence of nodes was obtained after running 1000 bootstrap replications.


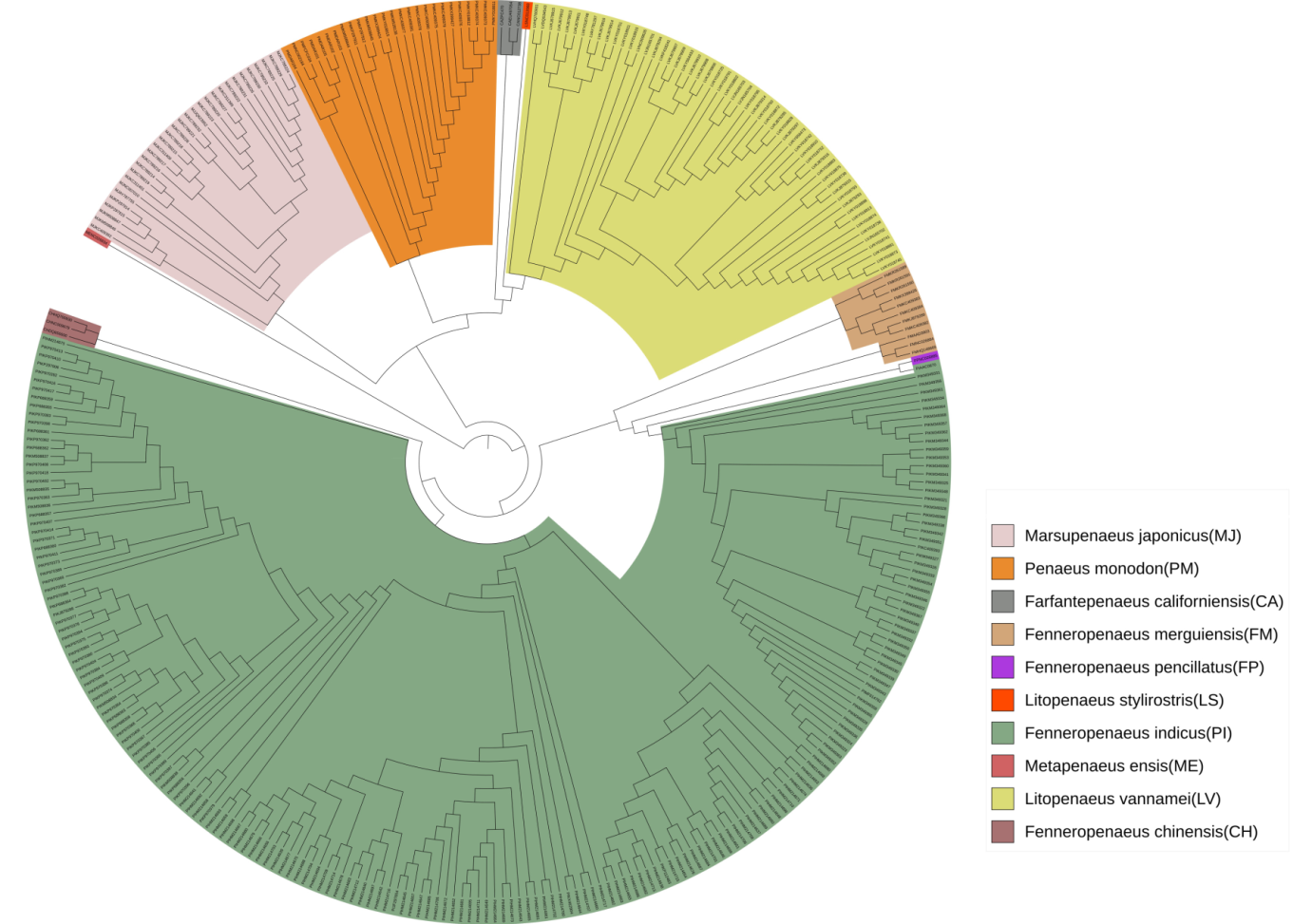


**Figure A1.** Maximum likelihood tree built with *F. indicus* accession under study and other *Penaeus* sensu lato species accessions from NCBI and BOLD database. The accession under study is clustered with other *F. indicus* accessions.

**Sequencing and assembly of mitochondrial DNA genome of *F. indicus***

The total DNA extracted from fresh muscle tissue was further processed to remove CpG-methylated nuclear DNA and enrich non-CpG-methylated mt DNA using NEBNext Microbiome DNA enrichment kit (NEB). The library of enriched DNA prepared using NEXTFlex DNA sequencing kit (Bioo Scientific), was subjected to paired-end sequencing on an Illumina NextSeq instrument. The quality of reads was assessed using fastqc_v0.11.5 [Andrews et al., 2010]. Poor quality reads (where the proportion of bases with Phred quality scores above 20 was less than 70 %) were filtered out and the poor quality bases (Phred quality score below 30) at 3’ ends of reads were trimmed using the perl scripts IlluQC.pl and TrimmingReads.pl respectively in NGSQC tool kit v2.3.3 [Patel et al., 2012]. Good quality reads were then assembled using the python-based assembler, SPAdes 3.7.1 [Nurk et al., 2013] and the contigs were further scaffolded using SSPACE 3.0 [Boetzer et al., 2011]. The annotation of protein-coding, rRNA and tRNA genes was obtained by DOGMA [Wyman et al., 2004], MITOS [Bernt et al., 2013] and tRNAscan-SE 1.21 [Lowe et al., 1997]. The circular visualization of mtDNA features was made in CGView server [Grant et al., 1998].

**Mitochondrial DNA genome of *F. indicus***

The specimen under study was observed to have morphological features specific to *F. indicus* like, short adrostral carina, closed thelycum and absence of gastro-frontal and hepatic carina. The study specimen was found to cluster along with other *F. indicus* accessions of NCBI and BOLD database as a separate clade in ML tree built with partial COI gene sequence. In this tree, the accessions of each species formed a separate clade. These morphological and molecular evidences confirm the species of the collected specimen as *F. indicus*.

For the first time, we deciphered the whole mitochondrial DNA genome of *F. indicus* with an aim to construct phylogenetic relations. The sequencing of library prepared with enriched DNA on Illumina platform generated 1,226,040 paired-reads. About 1,119,221 paired-reads that remained after removing poor quality reads were analysed in SPAdes 3.7.1 [Nurk et al., 2013] and SSPACE 3.0 [Boetzer et al., 2011] which produced a single scaffold of 16,071 bp. This genome sequence along with annotations has been deposited in GenBank (accession number KX462904).

The mtDNA genome of *F. indicus* is 16071 bp long and has 13 protein-coding, 2 rRNA and 22 tRNA genes similar to other penaeids (Table 1). The length-varying part of genome viz. the control region, is 1001 bp long and is located between *srRNA* and *tRNA^Ile^*. Twenty three genes are encoded by major (+) strand and the rest 14 genes by minor strand. Out of 13 protein-coding genes, nine are present on the heavy strand and four on the light strand. The ATG was the preferred start codon for majority of protein-coding genes (COX2, ATP6, COX3, ND3, ND5, ND4, ND4L AND CYTB) followed by ATT which initiates coding for ND2, ATP8 and ND6 genes. The ND1 and COX1 genes have ATA and ACG as start codons respectively. Only the start codon of COX1 gene could not be determined properly. Six genes have TAA and one gene has TAG as stop codon. The remaining six genes have incomplete stop codons which is common in animal mitochondrial genes. A 7 base overlap was observed between reading frames of ATP8 and ATP6 genes and also of ND4 and ND4L genes.

**Table A1. The sequence features of mitochondrial DNA genome of *Fenneropenaeus indicus*.**

| **Gene** | **feature** | **start** | **end** | **strand** |
| --- | --- | --- | --- | --- |
| **tRNA-Ile** | tRNA | 1 | 67 | + |
| **tRNA-Gln** | tRNA | 85 | 154 | - |
| **tRNA-Met** | tRNA | 186 | 253 | + |
| **ND2** | CDS | 254 | 1252 | + |
| **tRNA-Trp** | tRNA | 1254 | 1322 | + |
| **tRNA-Cys** | tRNA | 1410 | 1479 | - |
| **tRNA-Tyr** | tRNA | 1481 | 1545 | - |
| **COX1** | CDS | 1549 | 3084 | + |
| **tRNA-Leu** | tRNA | 3083 | 3148 | + |
| **COX2** | CDS | 3154 | 3840 | + |
| **tRNA-Lys** | tRNA | 3842 | 3910 | + |
| **tRNA-Asp** | tRNA | 3913 | 3980 | + |
| **ATP8** | CDS | 3981 | 4136 | + |
| **ATP6** | CDS | 4133 | 4801 | + |
| **COX3** | CDS | 4818 | 5606 | + |
| **tRNA-Gly** | tRNA | 5608 | 5673 | + |
| **ND3** | CDS | 5674 | 6024 | + |
| **tRNA-Ala** | tRNA | 6026 | 6090 | + |
| **tRNA-Arg** | tRNA | 6093 | 6157 | + |
| **tRNA-Asn** | tRNA | 6159 | 6227 | + |
| **tRNA-Ser** | tRNA | 6231 | 6297 | + |
| **tRNA-Glu** | tRNA | 6298 | 6367 | + |
| **tRNA-Phe** | tRNA | 6387 | 6454 | - |
| **ND5** | CDS | 6456 | 8177 | - |
| **tRNA-His** | tRNA | 8187 | 8253 | - |
| **ND4** | CDS | 8257 | 9594 | - |
| **ND4L** | CDS | 9591 | 9887 | - |
| **tRNA-Thr** | tRNA | 9890 | 9957 | + |
| **tRNA-Pro** | tRNA | 9958 | 10024 | - |
| **ND6** | CDS | 10026 | 10538 | + |
| **CYTB** | CDS | 10545 | 11678 | + |
| **tRNA-Ser** | tRNA | 11683 | 11752 | + |
| **ND1** | CDS | 11774 | 12709 | - |
| **tRNA-Leu** | tRNA | 12715 | 12781 | - |
| **l-rRNA** | rRNA | 12783 | 14136 | - |
| **tRNA-Val** | tRNA | 14149 | 14220 | - |
| **s-rRNA** | rRNA | 14222 | 15070 | - |

The arrangement of genes in mitochondrial genome is similar in all species of *Penaeus* sensu lato except for a minor change in *F. californiensis* wherein the position of tRNA-Thr was found to be altered. However, examination of the original manuscript related to complete mitochondrial DNA genome of *F. californiensis* [Peregrino-Uriarte et al., 2009] indicated that the gene arrangement is in fact similar to other shrimp species and it is only a typing error in Genbank accession.


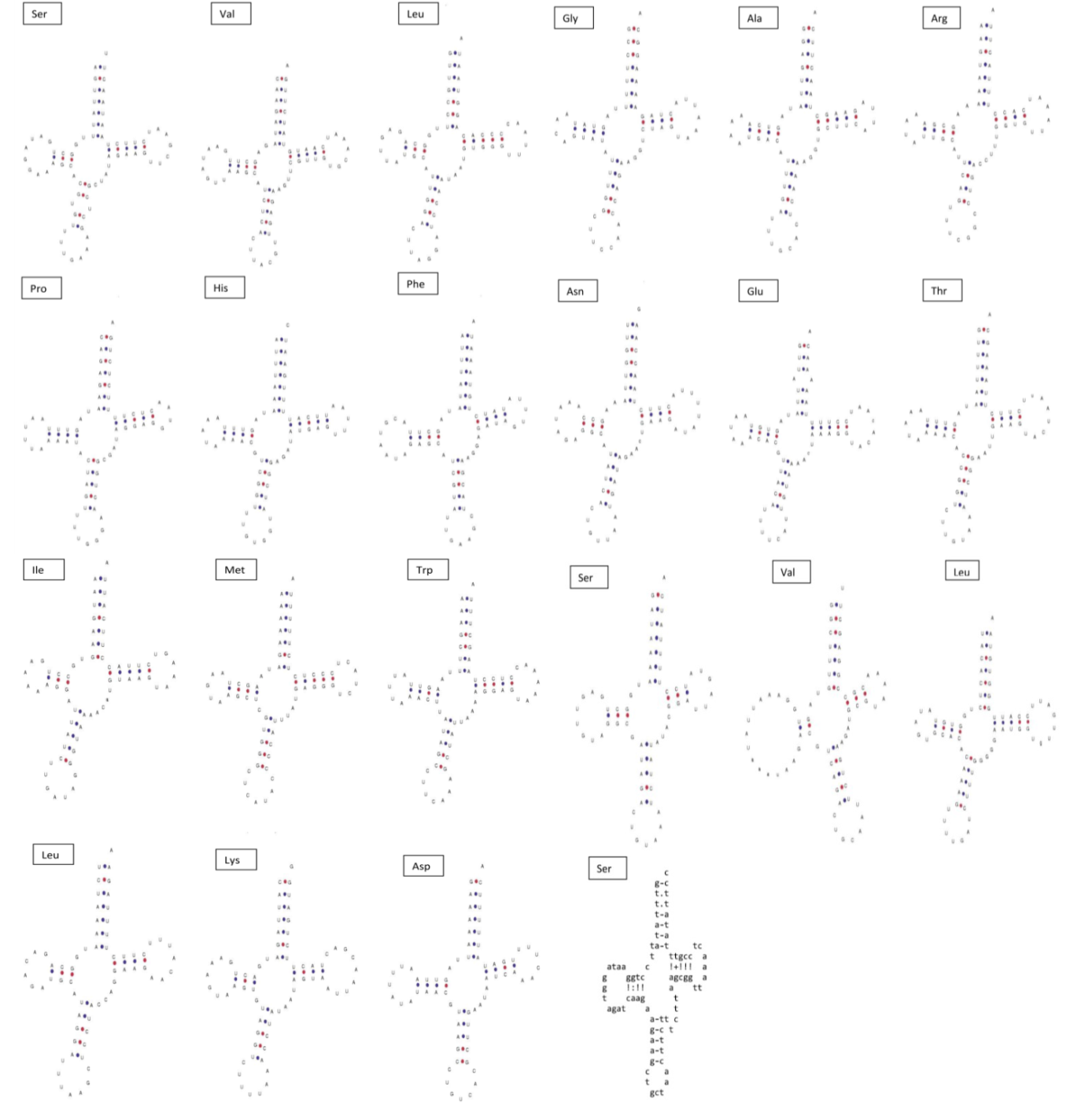


**Figure A2.** Secondary structures of tRNA genes in *F. indicus* mitochondrial DNA genome.

**References (Appendix A)**

1. Andrews, S. (2010). Babraham bioinformatics-FastQC a quality control tool for high throughput sequence data.  *Available at https://www.bioinformatics.babraham.ac.uk/projects/fastqc/.*
2. Bernt, M., Donath, A., Jühling, F., Externbrink, F., Florentz, C., Fritzsch, G., Putz, J., Middendorf, M. & Stadler, P. F. (2013). MITOS: improved de novo metazoan mitochondrial genome annotation. *Molecular phylogenetics and evolution*, **69**(2), 313-319.
3. Boetzer, M., Henkel, C. V., Jansen, H. J., Butler, D. & Pirovano, W. (2011). Scaffolding pre-assembled contigs using SSPACE. *Bioinformatics*, **27**(4), 578-579..
4. Botero-Castro, F., Delsuc, F., & Douzery, E. J. (2016). Thrice better than once: quality control guidelines to validate new mitogenomes. *Mitochondrial DNA Part A*, **27**(1), 449-454..
5. Grant, J. R., & Stothard, P. (2008). The CGView Server: a comparative genomics tool for circular genomes. Nucleic acids research, **36**(suppl_2), W181-W184.
6. Katoh, K., & Standley, D. M. (2013). MAFFT multiple sequence alignment software version 7: improvements in performance and usability. *Molecular biology and evolution*, **30**(4), 772-780.
7. Lowe, T. M. & Eddy, S. R. (1997). tRNAscan-SE: a program for improved detection of transfer RNA genes in genomic sequence. *Nucleic acids research*, **25**(5), 955-964.
8. Nurk, S., Bankevich, A., Antipov, D., Gurevich, A., Korobeynikov, A., Lapidus, A., Prjibelsky, A., Pyshkin, A., Sirotkin, A., Sirotkin, Y., Stepanauskas, R., Mclean, J., Lasken, R., Clingenpeel, S. R., Woyke, T., Tesler, G., Alekseyev, M. A. & Pevzner, P.A. (2013, April). Assembling genomes and mini-metagenomes from highly chimeric reads. In *Annual International Conference on Research in Computational Molecular Biology* (pp. 158-170). Springer, Berlin, Heidelberg.
9. Patel, R. K., & Jain, M. (2012). NGS QC Toolkit: a toolkit for quality control of next generation sequencing data. *PloS one*, **7**(2), e30619.
10. Peregrino-Uriarte, A. B., Varela-Romero, A., Muhlia-Almazán, A., Anduro-Corona, I., Vega-Heredia, S., Gutiérrez-Millán, L. E., Rosa-Velez, J. & Yepiz-Plascencia, G. (2009). The complete mitochondrial genomes of the yellowleg shrimp Farfantepenaeus californiensis and the blue shrimp Litopenaeus stylirostris (Crustacea: Decapoda). Comparative Biochemistry and Physiology Part D: Genomics and Proteomics, **4**(1), 45-53.
11. Sela, I., Ashkenazy, H., Katoh, K., & Pupko, T. (2015). GUIDANCE2: accurate detection of unreliable alignment regions accounting for the uncertainty of multiple parameters. *Nucleic acids research*, **43**(W1), W7-W14.
12. Stamatakis, A. (2014). RAxML version 8: a tool for phylogenetic analysis and post-analysis of large phylogenies. *Bioinformatics*, **30**(9), 1312-1313.
13. Wyman, S. K., Jansen, R. K. & Boore, J. L. (2004). Automatic annotation of organellar genomes with DOGMA. *Bioinformatics*, **20**(17), 3252-3255.

**APPENDIX B: Processing of sequence datasets**

This appendix contains the following sections which would help in replicating the analyses performed in this manuscript.

I. Alignment of sequence datasets (individual and concatenated alignment files can be accessed at [http://bioinfo.ciba.res.in/phylogeny_datasets/](http://bioinfo.ciba.res.in/phylogeny_supplementary/))

II. Results of analyses with Partition Finder (Finding best-fit models and partitioning schemes)

III. Bayesian analysis of sequence datasets

**I. Alignment of sequence datasets**

The study involved five different sequence alignments as listed below in the Table 1. The genus *Penaeus* sensu lato falls under suborder, Dendrobranchiata. About 29 species in this suborder have complete mtDNA genome information. Phylogenetic analysis was carried out with protein-coding and rRNA genes of these 29 accessions to document the phylogenetic position of *Penaeus* sensu lato relative to other accessions. Later, two additional accessions of the superfamily, Sergestoidea have been removed from the analysis retaining only one as outgroup (*S. lucens*). The phylogenetic relations were also established among the species in *Penaeus* sensu lato also.

**Table B1. Sequence datasets used for this study**.

| Sl. No. | Dataset | Number of accessions | Purpose |
| --- | --- | --- | --- |
| 1 | Concatenated rRNA genes | 11 mitochondrial genome accessions (10 species of *Penaeus* sensu lato and the *Metapenaeus ensis*) | Phylogenetic tree construction on Maximum likelihood and Bayesian principles |
| 2 | Concatenated Protein-coding genes |  |  |
| 3 | Concatenated rRNA genes | 29 mitochondrial genome accessions of suborder, Dendrobranchiata | Phylogenetic tree construction on Maximum likelihood and Bayesian principles |
| 4 | Concatenated Protein-coding genes |  |  |
| 5 | Cytochrome C Oxidase I gene | 328 accessions from BOLD database and NCBI | For molecular identification of *F. indicus* specimen under study |

1. **Alignment of rRNA genes (11 accessions):**

Initially, each type of rRNA gene sequences was aligned with MAFFT tool (Katoh and Standley, 2013) following L-INS-i strategy which is iterative refinement method incorporating local pairwise alignment information. The maximum iterations option was set to 1000. Individual rRNA gene alignments were also analysed using Guidance2 tool (Sela et al., 2015) to identify the positions in the alignment that have poor alignment confidence scores (< 0.93). The positions that were present in less than 25% of accessions (less than 3 in this case) were also removed. After removing all these positions (as detailed in Table B2), both the rRNA gene sequence alignments were concatenated to build the final alignment file that was used to build maximum likelihood and Bayesian trees.

**Table B2**. **Details of bases removed from rRNA genes before building concatenated alignment.**

|  | Positions removed | |  |  |
| --- | --- | --- | --- | --- |
| rRNA gene | Alignment confidence score, < 0.93 | Positions removed as they were present in <25 % of accessions | Positions remaining in final alignment | Base position in final concatenated alignment |
| 12S rRNA | 57-62, 75-85, 87-106, 109-112, 165, 198, 200, 284-294, 297-306, 308, 361-363, 432-441, 444-445, 567-570, 673-675, 698-699, 702-703, 725-733 | 43, 56, 108, 198-199, 283-284, 295-296, 307, 431, 566, 633-634, 700-701 | 763 | 1 – 763 |
| 16S rRNA | 3-39, 98-107, 158, 231-233, 376, 379-385, 387-389, 436, 438, 454-468, 528-535, 537, 539-544, 547-549, 552-553, 555-560, 568-570, 633, 637-649, 651-654, 682-683, 787-790, 1059-1065, 1067-1072, 1090, 1092-1098, 1100, 1369, 1372-1396, 1398-1435 | 1-2, 156-157, 376, 385-386, 463-464, 554, 634-636, 650, 680-681, 786, 1066, 1091, 1396-1399, 1436-1456 | 1201 | 764 - 1964 |

Files aligned with mafft: 12s_mafft_11Accessions.fasta; 16s_mafft_11Accessions.fasta

Final concatenated alignment: rRNA_concatenated_11Accessions.fasta

1. **Alignment of protein-coding genes (11 accessions):**

Initially, each protein-coding gene sequence was aligned with MAFFT tool (Katoh et al., 2005) following L-INS-i strategy with maximum iterations option set to 1000. Thereafter, positions in the alignment with low alignment confidence score as indicated by Guidance 2 tool were removed. Except in the case of ND5 gene, all positions in every other protein-coding gene have alignment confidence scores of more than 0.93. The detailed list of positions removed while making final alignment has been shown below in Table B3. The partitions of genes in final concatenated alignment are also shown in the Table B4.

**Table B3. Details of bases removed from protein-coding genes before building concatenated alignment.**

| Name of alignment made with MAFFT | Protein-coding gene | Positions removed | | | |
| --- | --- | --- | --- | --- | --- |
|  |  | Alignment confidence score, < 0.93 | Additional positions removed to keep codon structure | Bases of stop codon | Position present in less than 25% accessions |
| ATP6_mafft_11Accessions.fasta | ATP6 | - | - | 673-675 | - |
| ATP8_mafft_11Accessions.fasta | ATP8 | - | - | 157-159 | - |
| COX1_mafft_11Accessions.fasta | COX1 | - | - | 1534 | - |
| COX2_mafft_11Accessions.fasta | COX2 | - | - | 688 | - |
| COX3_mafft_11Accessions.fasta | COX3 | - | - | 790 | - |
| CYTB_mafft_11Accessions.fasta | CYTB | - | - | 1135-1137 | - |
| ND1_mafft_11Accessions.fasta | ND1 | - | - | 937-939 | - |
| ND2_mafft_11Accessions.fasta | ND2 | - | - | 1000-1002 | - |
| ND3_mafft_11Accessions.fasta | ND3 | - | - | 352 | - |
| ND4_mafft_11Accessions.fasta | ND4 | - | - | 1339-1341 | - |
| ND4L_mafft_11Accessions.fasta | ND4L | - | - | 298-300 | - |
| ND5_mafft_11Accessions.fasta | ND5 | 850-854, 902-907, 909 | 847, 848, 855, 856, 908, 910 | 1733-1735 | 1-9, 849, 1735 |
| ND6_mafft_11Accessions.fasta | ND6 | - | - | 514, 521, 522 | 515-520 |

**Table B4. Gene-wise partition in final concatenated file of protein-coding genes of *Penaeus* sensu lato accessions (proteincoding_concatenated_11Accessions.fasta):**

| Gene | Number of bases | Start position | End position |
| --- | --- | --- | --- |
| ATP6 | 672 | 1 | 672 |
| ATP8 | 156 | 673 | 828 |
| COX1 | 1533 | 829 | 2361 |
| COX2 | 687 | 2362 | 3048 |
| COX3 | 789 | 3049 | 3837 |
| CYTB | 1134 | 3838 | 4971 |
| ND1 | 936 | 4972 | 5907 |
| ND2 | 999 | 5908 | 6906 |
| ND3 | 351 | 6907 | 7257 |
| ND4 | 1338 | 7258 | 8595 |
| ND4L | 297 | 8596 | 8892 |
| ND5 | 1704 | 8893 | 10596 |
| ND6 | 513 | 10597 | 11109 |

1. **Alignment of rRNA genes from all complete mitochondrial genome accessions under suborder: Dendrobranchiata (29 accessions):**

The strategy adopted for making concatenated alignment for 11 accessions as listed above in point number 1, was also followed for making alignments of rRNA genes involving 29 accessions. The positions that were present in less than 25% of accessions (less than 8 in this case) were also removed. After removing all these positions (as detailed in Table B5), both the rRNA gene sequence alignments were concatenated to build the final alignment file that was used to build maximum likelihood and Bayesian trees.

**Table B5. Details of bases removed from rRNA genes before building concatenated alignment.**

|  | Positions removed | |  |  |
| --- | --- | --- | --- | --- |
| rRNA gene | Alignment confidence score, < 0.93 | Positions removed as they were present in <25 % of accessions | Positions remaining in final alignment | Base position in final concatenated alignment |
| 12S rRNA | 31-32, 34-55, 68-144, 178-181, 214-220, 239, 241-242, 308-323, 326-335, 364-367, 377-418, 475-482, 484-486, 606-610, 612-613, 647, 654-657, 674-677, 679-680, 714-719, 741-747, 749-769, 772-782, 873, 929-930 | 1-9, 33, 182, 183, 221-224, 238, 272, 324, 325, 368-376, 483, 568, 581, 611, 678, 748, 770, 771, 808, 828, 931 | 627 | 1 – 627 |
| 16S rRNA | 19-110, 127-141, 143-154, 163-165, 188-190, 192-205, 262-280, 306-307, 310-315, 317-318, 378-382, 417-467, 483, 491-497, 510-533, 584-613, 615-656, 658-666, 691-698, 704-717, 719-754, 757-768, 787-792, 831-833, 865-872, 1061-1084, 1087-1104, 1141-1143, 1145-1163, 1165-1194, 1427-1429, 1431-1437, 1482-1485, 1487-1552 | 1-18, 111-126, 142, 162, 191, 308, 309, 377, 484, 498-509, 614, 699-703, 718, 755, 756, 769, 786, 829, 830, 891, 971, 1060, 1085, 1086, 1109, 1110, 1144, 1164, 1195, 1261, 1275-1287, 1345, 1400, 1430, 1465-1467, 1486, 1553-1567 | 857 | 628 - 1484 |

Files aligned with mafft: 12s_mafft_29Accessions.fasta; 16s_mafft_29Accessions.fasta

Final concatenated alignment: rRNA_concatenated_29Accessions.fasta

1. **Alignment of protein-coding genes from all complete mitochondrial genome accessions under suborder: Dendrobranchiata (29 accessions):**

The strategy adopted for making concatenated alignment for 11 accessions as listed above in point number 2, was also followed for making alignments of protein-coding genes involving 29 accessions. The details are listed in Table B6 below. The partitions of genes in final concatenated alignment are also shown in the Table B7.

**Table B6. Details of bases removed from protein-coding genes before building concatenated alignment involving 29 accessions.**

| File name | Protein-coding gene | Positions removed | | | |
| --- | --- | --- | --- | --- | --- |
|  |  | Alignment confidence score, < 0.93 | Additional positions removed to keep codon structure | Bases of stop codon | Position present in less than 25% accessions |
| ATP6_mafft_29Accessions.fasta | ATP6 | - | - | 673-675 | - |
| ATP8_mafft_29Accessions.fasta | ATP8 | - | - | 157-159 | - |
| COX1_mafft_29Accessions.fasta | COX1 | - | - | 1540 | 1-6, 1541-1545 |
| COX2_mafft_29Accessions.fasta | COX2 | - | - | 688 | 689-708 |
| COX3_mafft_29Accessions.fasta | COX3 | - | - | 791 | 763, 792-838 |
| CYTB_mafft_29Accessions.fasta | CYTB | - | - | 1135-1137 | - |
| ND1_mafft_29Accessions.fasta | ND1 | 939 | 938, 940 | 944-946 | 1-3, 920, 941-943 |
| ND2_mafft_29Accessions.fasta | ND2 | 930-940 | 928-929, 941-942 | 1032-1034 | 428-430, 911-913, 952-977 |
| ND3_mafft_29Accessions.fasta | ND3 | 353, 354 | - | 352 | 355-405 |
| ND4_mafft_29Accessions.fasta | ND4 | 1317-1320, 1324 | 1315-1316, 1325-1326 | 1339-1341 | - |
| ND4L_mafft_29Accessions.fasta | ND4L | - | - | 309-311 | 20-30 |
| ND5_mafft_29Accessions.fasta | ND5 | - | - | 1742-1744 | 853, 1579-1587 |
| ND6_mafft_29Accessions.fasta | ND6 | 267, 272-303, 352-392 | 256, 257, 271 | 540-542 | 258-266, 304-306, 533-538 |

**Table B7. Gene-wise partition in final concatenated file of protein-coding genes of 29 complete mtDNA genome accessions under suborder Dendrobranchiata (Protein-coding_concatenated_29accessions.fasta):**

| Gene | Number of bases | Start position | End position |
| --- | --- | --- | --- |
| ATP6 | 672 | 1 | 672 |
| ATP8 | 156 | 673 | 828 |
| COX1 | 1533 | 829 | 2361 |
| COX2 | 687 | 2362 | 3048 |
| COX3 | 789 | 3049 | 3837 |
| CYTB | 1134 | 3838 | 4971 |
| ND1 | 933 | 4972 | 5904 |
| ND2 | 984 | 5905 | 6888 |
| ND3 | 351 | 6889 | 7239 |
| ND4 | 1329 | 7240 | 8568 |
| ND4L | 297 | 8569 | 8865 |
| ND5 | 1731 | 8866 | 10596 |
| ND6 | 444 | 10597 | 11040 |

1. **Alignment of COX1 gene:**

About 326 COX1 gene accessions that belong to species under the genus *Penaeus* sensu lato have been downloaded from BOLD database (<http://www.boldsystems.org/>). Two other sequences of COX1 gene, one that of the *Fenneropenaeus indicus* specimen whose mitochondrial genome was sequenced in this study (KX462904) and the other an accession of *Metapenaeus ensis* from BOLD database which is included as outgroup, have been included in final alignment. The alignment of 328 sequences of COX 1 gene was made with MAFFT tool. Extracting only the consensus part of the alignment has reduced the sequence length to 531 bases. All the positions have high alignment confidence scores (>0.93) as generated using Guidance2 tool. This alignment was used to build a maximum likelihood tree using RAxML tool.

Final alignment: COX1_BOLD.fasta

**II. Results of analyses with Partition Finder**

The tool PartitionFinder 2 (Lanfear et al., 2017) was used to find the best evolutionary model and the best partitioning scheme for the sequence data used in this study. Some of the common settings used for analysis are linked branch lengths and greedy search. The best model was selected based on the Akaike information criterion (AIC).

The RAxML tool (Stamatakis, 2014) used for building maximum likelihood tree in this study can use only one model for all the partitions. Therefore, partition finder tool is run to find the best partition scheme for GTRGAMMAI model. The MrBayes tool (Huelsenbeck, 2001) that was used for building Bayesian trees in the study can model different models for different partitions. Therefore the models chosen as best for each partition were used to build Bayesian trees.

1. **Protein-coding genes (29 accessions of Suborder Dendrobranchiata):**

**1.1. For building RAxML tree:**

Best partitioning scheme:

Scheme Name : step_18

Scheme lnL : -148034.83032226562

Scheme AICc : 296659.035645

Number of params : 287

Number of sites : 11040

Number of subsets : 21

| Subset | # sites | Partition names |
| --- | --- | --- |
| 1 | 341 | ATP6_pos1, ND3_pos1 |
| 2 | 341 | ATP6_pos2, ND3_pos2 |
| 3 | 552 | ND2_pos3, ATP6_pos3 |
| 4 | 528 | ATP8_pos1, ND2_pos1, ND6_pos1 |
| 5 | 528 | ND6_pos2, ND2_pos2, ATP8_pos2 |
| 6 | 200 | ATP8_pos3, ND6_pos3 |
| 7 | 1118 | CYTB_pos1, COX1_pos1, COX2_pos1 |
| 8 | 511 | COX1_pos2 |
| 9 | 740 | COX1_pos3, COX2_pos3 |
| 10 | 229 | COX2_pos2 |
| 11 | 263 | COX3_pos1 |
| 12 | 641 | CYTB_pos2, COX3_pos2 |
| 13 | 641 | CYTB_pos3, COX3_pos3 |
| 14 | 311 | ND1_pos1 |
| 15 | 311 | ND1_pos2 |
| 16 | 311 | ND1_pos3 |
| 17 | 117 | ND3_pos3 |
| 18 | 1119 | ND5_pos1, ND4_pos1, ND4L_pos1 |
| 19 | 542 | ND4L_pos2, ND4_pos2 |
| 20 | 1119 | ND4L_pos3, ND4_pos3, ND5_pos3 |
| 21 | 577 | ND5_pos2 |

**1.2.For building Bayesian tree:**

Best partitioning scheme

Scheme Name : step_15

Scheme lnL : -148008.31182861328

Scheme AICc : 296633.440866

Number of params : 300

Number of sites : 11040

Number of subsets : 24

| Subset | Best Model | # sites | Partition names |
| --- | --- | --- | --- |
| 1 | GTR+I+G | 341 | ATP6_pos1, ND3_pos1 |
| 2 | GTR+I+G | 341 | ATP6_pos2, ND3_pos2 |
| 3 | GTR+I+G | 372 | ATP6_pos3, ND6_pos3 |
| 4 | HKY+G | 52 | ATP8_pos1 |
| 5 | GTR+I+G | 528 | ND6_pos2, ND2_pos2, ATP8_pos2 |
| 6 | HKY+G | 52 | ATP8_pos3 |
| 7 | GTR+I+G | 740 | COX1_pos1, COX2_pos1 |
| 8 | GTR+I+G | 511 | COX1_pos2 |
| 9 | GTR+I+G | 740 | COX1_pos3, COX2_pos3 |
| 10 | GTR+G | 229 | COX2_pos2 |
| 11 | GTR+I+G | 263 | COX3_pos1 |
| 12 | GTR+I+G | 641 | CYTB_pos2, COX3_pos2 |
| 13 | GTR+I+G | 641 | CYTB_pos3, COX3_pos3 |
| 14 | SYM+I+G | 378 | CYTB_pos1 |
| 15 | GTR+I+G | 311 | ND1_pos1 |
| 16 | GTR+I+G | 311 | ND1_pos2 |
| 17 | GTR+I+G | 311 | ND1_pos3 |
| 18 | GTR+I+G | 476 | ND6_pos1, ND2_pos1 |
| 19 | GTR+G | 328 | ND2_pos3 |
| 20 | HKY+G | 117 | ND3_pos3 |
| 21 | GTR+I+G | 1119 | ND5_pos1, ND4L_pos1, ND4_pos1 |
| 22 | GTR+I+G | 542 | ND4_pos2, ND4L_pos2 |
| 23 | GTR+I+G | 1119 | ND4L_pos3, ND4_pos3, ND5_pos3 |
| 24 | GTR+I+G | 577 | ND5_pos2 |

**2. Protein-coding genes (11 accessions of *Penaeus* sensu lato):**

**2.1. For building RAxML tree:**

Best partitioning scheme:

Scheme Name : step_21

Scheme lnL : -55414.9873046875

Scheme AICc : 111270.581293

Number of params : 216

Number of sites : 11109

Number of subsets : 18

| Subset | # sites | Partition names |
| --- | --- | --- |
| 1 | 674 | ND2_pos1, ATP6_pos1, ND3_pos1 |
| 2 | 341 | ATP6_pos2, ND3_pos2 |
| 3 | 395 | ATP6_pos3, ND6_pos3 |
| 4 | 223 | ATP8_pos1, ND6_pos1 |
| 5 | 556 | ND6_pos2, ND2_pos2, ATP8_pos2 |
| 6 | 1055 | COX2_pos3, COX3_pos3, COX1_pos3, ATP8_pos3 |
| 7 | 1118 | COX1_pos1, COX2_pos1, CYTB_pos1 |
| 8 | 1052 | COX2_pos2, COX1_pos2, ND1_pos2 |
| 9 | 263 | COX3_pos1 |
| 10 | 641 | COX3_pos2, CYTB_pos2 |
| 11 | 495 | CYTB_pos3, ND3_pos3 |
| 12 | 411 | ND1_pos1, ND4L_pos1 |
| 13 | 880 | ND1_pos3, ND5_pos3 |
| 14 | 333 | ND2_pos3 |
| 15 | 1014 | ND5_pos1, ND4_pos1 |
| 16 | 545 | ND4_pos2, ND4L_pos2 |
| 17 | 545 | ND4L_pos3, ND4_pos3 |
| 18 | 568 | ND5_pos2 |

**2.2. For building Bayesian tree:**

Best partitioning scheme:

Scheme Name : step_18

Scheme lnL : -55391.554443359375

Scheme AICc : 111221.635463

Number of params : 215

Number of sites : 11109

Number of subsets : 21

| Subset | Best Model | # sites | Partition names |
| --- | --- | --- | --- |
| 1 | GTR+G | 674 | ND2_pos1, ATP6_pos1, ND3_pos1 |
| 2 | GTR+I | 341 | ATP6_pos2, ND3_pos2 |
| 3 | GTR+I+G | 395 | ND6_pos3, ATP6_pos3 |
| 4 | HKY+I | 223 | ND6_pos1, ATP8_pos1 |
| 5 | GTR+I+G | 556 | ND6_pos2, ND2_pos2, ATP8_pos2 |
| 6 | HKY+G | 52 | ATP8_pos3 |
| 7 | GTR+G | 511 | COX1_pos1 |
| 8 | F81+I | 740 | COX1_pos2, COX2_pos2 |
| 9 | GTR+I+G | 511 | COX1_pos3 |
| 10 | SYM+I | 607 | COX2_pos1, CYTB_pos1 |
| 11 | GTR+I+G | 492 | COX3_pos3, COX2_pos3 |
| 12 | SYM+I | 263 | COX3_pos1 |
| 13 | GTR+I | 641 | COX3_pos2, CYTB_pos2 |
| 14 | GTR+I+G | 495 | CYTB_pos3, ND3_pos3 |
| 15 | GTR+I+G | 411 | ND1_pos1, ND4L_pos1 |
| 16 | HKY+I | 411 | ND1_pos2, ND4L_pos2 |
| 17 | GTR+I+G | 880 | ND1_pos3, ND5_pos3 |
| 18 | GTR+G | 333 | ND2_pos3 |
| 19 | GTR+I+G | 1014 | ND4_pos1, ND5_pos1 |
| 20 | GTR+I+G | 1014 | ND5_pos2, ND4_pos2 |
| 21 | GTR+I+G | 545 | ND4L_pos3, ND4_pos3 |

**3. COX1 gene used for molecular identification of Indian white shrimp specimen:**

Best partitioning scheme

Scheme Name : step_2

Scheme lnL : -4773.765625

Scheme AICc : 888683.53125

Number of params : 662

Number of sites : 531

Number of subsets : 1

| Subset | Best Model | # sites | Partition names |
| --- | --- | --- | --- |
| 1 | GTR+G+X | 531 | Cox1_pos1, Cox1_pos2, Cox1_pos3 |

**III. Bayesian analysis of sequence datasets**

All the Bayesian analysis runs were done using MrBayes v 3.2.6 tool in a tower work station (Intel Xeon 3.30 GHz processor, 32 GB RAM, 64-bit operating system). Sequence files in ‘nexus’ formats were used in analysis. For every dataset, two simultaneous but completely independent runs that start from different random trees were executed with 4 chains for ten million generations while sampling trees every 100 generations and calculating convergence statistics every 1000 generations. First 25% of samples were discarded and not included for calculating summary statistics. The maximum standard deviation of split frequencies and potential scale reduction factor, PSRF (Gelman and Rubin, 1992) were used to check the convergence of runs. The same was performed by also examining the trace files in Tracer v1.6 [Rambaut A 2018].

**1. Protein-coding genes dataset (29 accessions of Dendrobranchiata):**

The accession of *Belzebub intermedius* was used as outgroup.

Seed (for generating default start values) = 1550923590

Number of character partitions = 24

Seed (for generating default start values) while defining partition= 2111847524

Model used: For each data partition, the best model as indicated in the results of partition finder was defined.

Maximum standard deviation of split frequencies after 10 million generations: 0.000933

Analysis completed in 17 hours 35 minutes and 44 seconds.

Analysis used 63343.67 seconds of CPU time

Summary statistics:

Average standard deviation of split frequencies = 0.000035

Average PSRF for parameter values = 1.017

Maximum PSRF for parameter values = 1.033

**2. rRNA genes dataset (29 accessions of Dendrobranchiata):**

The accession of *Belzebub intermedius* was used as outgroup.

Seed (for generating default start values) = 1550032626

Number of character partitions = 2

Seed (for generating default start values) while defining partition= 915381437

Maximum standard deviation of split frequencies after 10 million generations: 0.004082

Analysis completed in 4 hours 34 minutes and 38 seconds.

Analysis used 16477.05 seconds of CPU time

Summary statistics:

Average standard deviation of split frequencies = 0.000909

Average PSRF for parameter values = 1.000

Maximum PSRF for parameter values = 1.000

**References (Appendix B):**

1. Gelman, A. & Rubin, D. B. (1992). Inference from iterative simulation using multiple sequences. *Statistical science*, **7**(4), 457-472.
2. Huelsenbeck, J. P. & Ronquist, F. (2001). MRBAYES: Bayesian inference of phylogenetic trees. *Bioinformatics*, **17**(8), 754-755.
3. Katoh, K. & Standley, D. M. (2013). MAFFT multiple sequence alignment software version 7: improvements in performance and usability. *Molecular biology and evolution*, **30**(4), 772-780.
4. Lanfear, R., Frandsen, P. B., Wright, A. M., Senfeld, T. & Calcott, B. (2017). PartitionFinder 2: new methods for selecting partitioned models of evolution for molecular and morphological phylogenetic analyses. *Molecular biology and evolution*, **34**(3), 772-773.
5. Rambaut, A., Drummond, A. J., Xie, D., Baele, G. & Suchard, M. A. Tracer v1.7. 2018 Available from http://tree.bio.ed.ac.uk/software/tracer.
6. Sela, I., Ashkenazy, H., Katoh, K. & Pupko, T. (2015). GUIDANCE2: accurate detection of unreliable alignment regions accounting for the uncertainty of multiple parameters. *Nucleic acids research*, **43**(W1), W7-W14.
7. Stamatakis, A. (2014). RAxML version 8: a tool for phylogenetic analysis and post-analysis of large phylogenies. *Bioinformatics*, **30**(9), 1312-1313.

**APPENDIX C: Average Aminoacid Identity estimates among species of each Genus and among genera of each Family in the order, Decapoda**


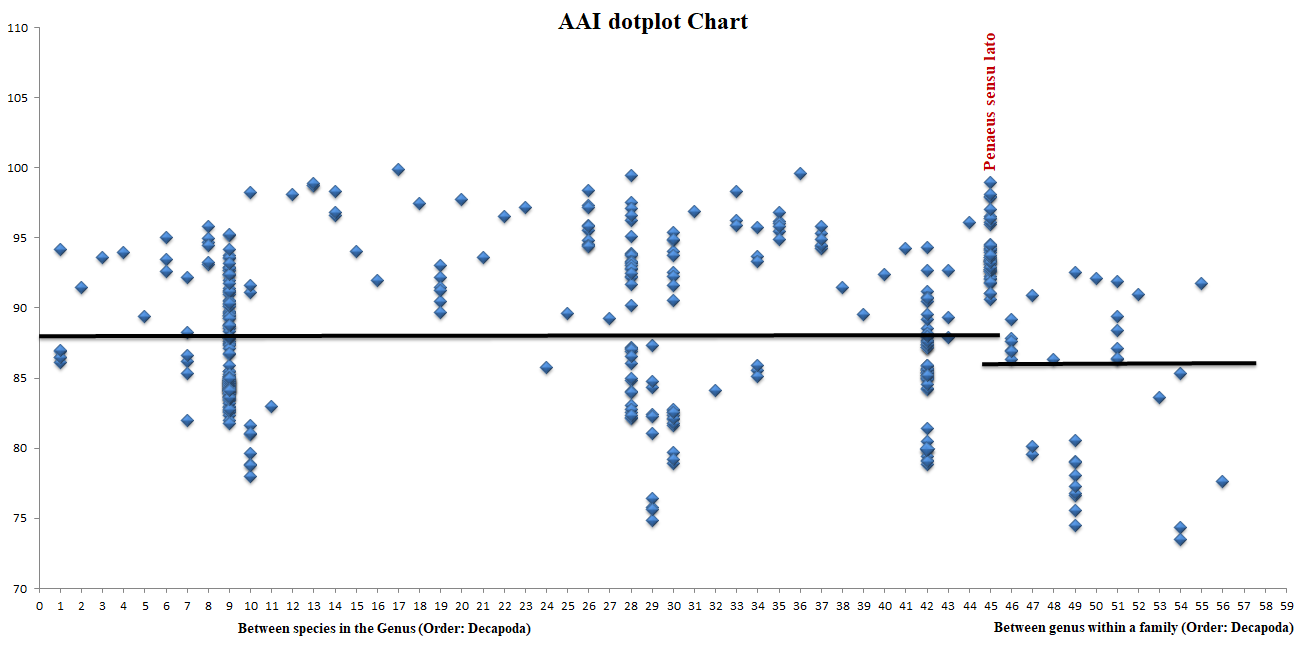


Figure C1. A dotplot chart of AAI estimates obtained for genera and families in the order, Decapoda. The horizontal line in the plot separates the highest 50% and the lowest 50% AAI estimates. All the estimates in the genus, *Penaeus* sensu lato are in the highest 50% estimates. The labels of the X-axis are, 1=Genus, Alpheus; 2=Genus, Alvinocaris; 3=Genus, Atergatis; 4=Genus, Austinograea; 5=Genus, Austropotamobius; 6=Genus, Cambaroides; 7=Genus, Caridina; 8=Genus, Charybdis; 9=Genus, Cherax; 10=Genus, Engaeus; 11=Genus, Engaewa; 12=Genus, Enoplometopus; 13=Genus, Eriocheir; 14=Genus, Euastacus; 15=Genus, Gandalfus; 16=Genus, Gramastacus; 17=Genus, Helice; 18=Genus, Homarus; 19=Genus, Macrobrachium; 20=Genus, Maja; 21=Genus, Metanephrops; 22=Genus, Metapenaeopsis; 23=Genus, Metapenaeus; 24=Genus, Munida; 25=Genus, Ocypode; 26=Genus, Orconectes; 27=Genus, Pachygrapsus; 28=Genus, Pagurus; 29=Genus, Palaemon; 30=Genus, Panulirus; 31=Genus, Paralithodes; 32=Genus, Parapenaeopsis; 33=Genus, Parasesarama; 34=Genus, Portunus; 35=Genus, Procambarus; 36=Genus, Rimicaris; 37=Genus, Scylla; 38=Genus, Sicyonia; 39=Genus, Stygiocaris; 40=Genus, Thalamita; 41=Genus, Tubuca; 42=Genus, Typhlatya; 43=Genus, Upogebia; 44=Genus, Xenograpsus; 45=Genus, Penaeus; 46=Family, Penaeidae; 47=Family, Cambaridae; 48=Family, Nephropidae; 49=Family, Parastacidae; 50=Family, Bythograeidae; 51=Family, Portunidae; 52=Family, Varunidae; 53=Family, Ocypodidae; 54=Family, Atyidae; 55=Family, Alvinocarididae; 56=Family, Palaemonidae

**APPENDIX D: Ranges of between-species Average Aminoacid Identity (AAI) in different genera under the order, Decapoda.**

| **Sl.**  **No.** | **Genus** | **No. of**  **species** | **Ranges of**  **AAI** | **Accessions** |
| --- | --- | --- | --- | --- |
| 1 | Alpheus | 4 | 86.08 – 94.20 | GQ892049, MG873459, MG787409, KP276147 |
| 2 | Alvinocaris | 2 | 91.49 | JX184903, JQ035659 |
| 3 | Atergatis | 2 | 93.58 | MG792341, MG786939 |
| 4 | Austinograea | 2 | 93.97 | JQ035660, JQ035658 |
| 5 | Austropotamobius | 2 | 89.40 | KP205430, KX268734 |
| 6 | Cambaroides | 3 | 92.58 – 95.04 | KX268735, KX268736, JN991196 |
| 7 | Caridina | 4 | 82.00 – 92.14 | KM023648, MH189850, MG580781, KU726823 |
| 8 | Charybdis | 4 | 93.11 - 95.80 | MG489891, KF386147, FJ460517, MF285241 |
| 9 | Cherax | 17 | 81.76 - 95.27 | KM501041, KM501042, KF649849, HG799094,  HG942365, AY383557, HG799093, KF649852,  HG799096, KX119167, KM501039, KF649851,  HG799097, KF649850, HG799091, HG799090,  KP205429 |
| 10 | Engaeus | 5 | 77.95 - 98.22 | HG942173, KF546209, HG799086, LK391949,  LK391948 |
| 11 | Engaewa | 2 | 82.97 | KT946764, KT946765 |
| 12 | Enoplometopus | 2 | 98.11 | KM488333, KC107819 |
| 13 | Eriocheir | 3 | 98.71 - 98.87 | FJ455506, FJ455505, AY274302 |
| 14 | Euastacus | 3 | 96.63 - 98.33 | KP294310, KM458972, HG942176 |
| 15 | Gandalfus | 2 | 94.03 | KR002727, EU647222 |
| 16 | Gramastacus | 2 | 91.95 | KX148478, LK022684 |
| 17 | Helice | 2 | 99.87 | KU589291, KR336555 |
| 18 | Homarus | 2 | 97.46 | HQ402925, KC107810 |
| 19 | Macrobrachium | 4 | 89.68 – 93.00 | KM978918, AY659990, HQ830201, FJ797435 |
| 20 | Maja | 2 | 97.72 | KY650651, KY650652 |
| 21 | Metanephrops | 2 | 93.58 | LN611668, KP889215 |
| 22 | Metapenaeopsis | 2 | 96.5 | KU050082, MG833230 |
| 23 | Metapenaeus | 2 | 97.2 | MG815825, KP637170 |
| 24 | Munida | 2 | 85.74 | KU521508, MF457406 |
| 25 | Ocypode | 2 | 89.61 | LN611669, MG787409 |
| 26 | Orconectes | 5 | 94.37 - 98.36 | KP205431, KX268739, KX119150, KU239994,  KU239995 |
| 27 | Pachygrapsus | 2 | 89.26 | KC878511, MF457403 |
| 28 | Pagurus | 9 | 82.13 – 99.42 | LC222528, LC222534, LC222532, LC222527,  AF150756, LC222524, LC222533, LC222531,  LC222535 |
| 29 | Palaemon | 5 | 74.84 – 87.30 | FJ797435, FJ797435, KT935323, MF687349,  KM978916 |
| 30 | Panulirus | 7 | 78.93 - 95.40 | MH068821, KT696496, JN542716, AB071201,  GQ223286, GQ292768, KC107808 |
| 31 | Paralithodes | 2 | 96.90 | AB735677, JX944381 |
| 32 | Parapenaeopsis | 2 | 84.13 | KU302814, MG873460 |
| 33 | Parasesarma | 3 | 95.91 – 98.28 | MH310444, MG580780, KU343209 |
| 34 | Portunus | 4 | 85.09 - 95.72 | MH729187, KM977882, KT438509, AB093006 |
| 35 | Procambarus | 4 | 95.44 - 96.79 | KC107813, KX268741, KT074363, JN991197 |
| 36 | Rimicaris | 2 | 99.57 | KP284529, JQ035656 |
| 37 | Scylla | 4 | 94.21 - 95.83 | FJ827760, FJ827761, FJ827758, FJ827759 |
| 38 | Sicyonia | 2 | 91.48 | MF379619, MF379620 |
| 39 | Stygiocaris | 2 | 89.55 | KX844722, KX844714 |
| 40 | Thalamita | 2 | 92.38 | LK391945, MG840650 |
| 41 | Tubuca | 2 | 94.21 | MF457401, MF457400 |
| 42 | Typhlatya | 11 | 79.03 - 94.31 | KX844721, KX844717, KX844719, KX844711,  KX844720, KX844710, LT608343, KX844712,  KX844715, KX844709, KX844708 |
| 43 | Upogebia | 3 | 87.86 - 92.70 | KM886610, JN897377, KC107815 |
| 44 | Xenograpsus | 2 | 96.10 | KY985236, EU727203 |
| 45 | *Penaeus* sensu lato | 10 | 90.57 – 98.95 | EU497054, DQ518969, KX462904, KP637168,  KP637169, EU517503, EF584003, AP006346,  MG821353, AF217843 |

**APPENDIX E. Phylogenetic relations among the shrimp in the genus, *Penaeus* sensu lato.**


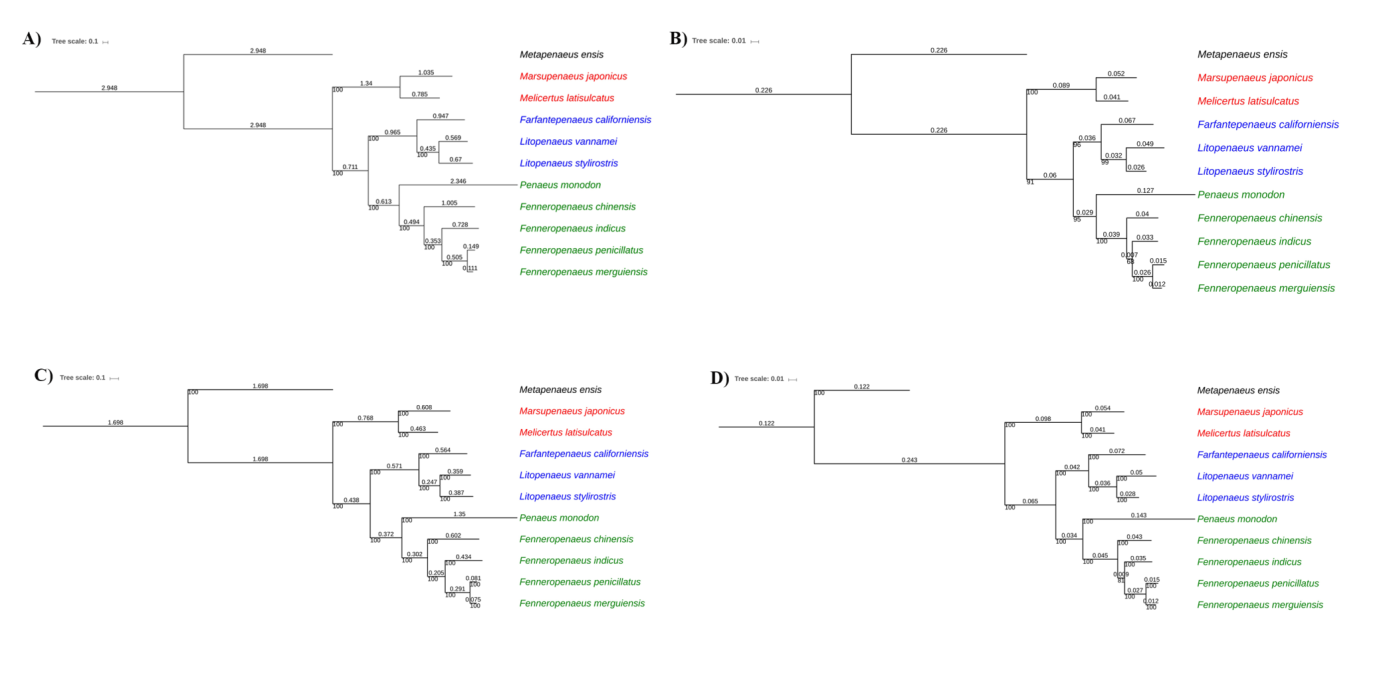


**Figure D1.** Maximum likelihood tree for A) protein coding genes and B) rRNA genes & Bayes tree for C) protein coding genes and D) rRNA genes for species in *Penaeus* sensu lato. The *Metpenaeus ensis* is the outgroup.
